# Enhancing CO_2_ Reduction Performance on Cu-Based Catalysts: Modulating Electronic Properties and Molecular Configurations

**DOI:** 10.3390/ma18214964

**Published:** 2025-10-30

**Authors:** Huimin Han, Luxin Yang, Chao Han, Maosheng Bi, Hongbo Li, Yuwei Zeng, Kunming Pan, Shengyu Yin, Fang Wang, Saifei Pan

**Affiliations:** 1 School of Materials Science and Engineering, Henan University of Science and Technology, Luoyang 471000, China; 2 School of Energy and Chemical Engineering, Luoyang Institute of Science and Technology, Luoyang 471023, China; 3 Longmen Laboratory, Luoyang 471000, China; 4 Longbai Group Co., Ltd., Jiaozuo 454191, China; 5 Henan Key Laboratory of Green Building Materials Manufacturing and Intelligent Equipment, Luoyang 471023, China; 6 College of Vehicle and Traffic Engineering, Henan University of Science and Technology, Luoyang 471003, China

**Keywords:** carbon dioxide reduction reaction, Cu-based catalyst, electronic effect, structural tuning, selectivity

## Abstract

**Highlights:**

**What are the main findings?**

- Summarizes unique advantages and catalytic mechanisms of Cu-based catalysts.
- Systematically elaborates three tuning strategies for Cu-based catalysts.
- Identifies core challenges of Cu-based catalysts in CO_2_RR.

**What are the implications of the main findings?**

- Providing a theoretical framework for the rational design of Cu-based catalysts.
- Regulating Cu active site properties and intermediate adsorption behavior.
- Lays a foundation for advancing efficient CO_2_RR catalytic systems and practical application.

**Abstract:**

The renewable-energy-powered electrocatalytic CO_2_ reduction reaction (CO_2_RR) efficiently converts CO_2_ into high-value chemicals and fuels, offering a promising approach to addressing environmental and energy sustainability challenges. This process is of immense significance for constructing a sustainable artificial carbon cycle. Cu-based catalysts exhibit remarkable catalytic activity and broad product selectivity in CO_2_RR, which can be attributed to their excellent electrical conductivity, moderate adsorption energy, and unique electronic structure. This review comprehensively summarizes the advantages, practical applications, and mechanistic insights of Cu-based catalysts in CO_2_RR, with a systematic based on recent advances in tuning strategies via electronic effects and structural design. Specifically, it emphasizes the influence of electronic structure tuning (electron-donating/-withdrawing effects and steric hindrance effects), active center tuning (single-atom catalysts, heterogeneous synergetic effects, and polymer modification), and surface structure (morphology effect, valence-state effect, and crystalline-facet effect) influences on catalytic performance. By rationally designing the catalyst structure, the adsorption behavior of reaction intermediates can be effectively regulated, thereby enabling the highly selective generation of target products. The objective of this paper is to provide a theoretical framework and actionable strategies for the structural design and catalytic performance optimization of Cu-based catalysts, with the ultimate goal of promoting the development and practical application of efficient CO_2_RR catalytic systems.

## 1. Introduction

The accelerated pace of global industrialization, along with sustained population expansion and extensive consumption of energy fuels, has led to a substantial increase in greenhouse gas emissions. This trend has now become a critical global challenge that requires immediate and coordinated international efforts to address. Among these, carbon dioxide (CO_2_) is the most predominant one. Its elevated concentration severely disrupts the global carbon cycle and is widely regarded as a primary driver of anthropogenic global climate change [1,2]. To tackle this issue, achieving carbon neutrality has been established as the core objective of global environmental governance, which is essential for sustaining the long-term development of human society. Technologies capable of effectively converting CO_2_ into high-value chemicals or sustainable fuels are considered critical approaches for closing the carbon cycle and fulfilling carbon neutrality goals [3].

Among various CO_2_ conversion methods, the electrocatalytic reduction of carbon dioxide has attracted significant attention due to its compatibility with renewable electricity, feasibility of operation under ambient conditions, and ability to efficiently produce valuable carbon-containing compounds. These include C_1_ species, C_2_ products, and other multi-carbon compounds [4,5,6,7]. The core mechanism of CO_2_RR involves two key steps: first, CO_2_ molecules are adsorbed at the electrode–electrolyte interface; then, proton-coupled electron transfer (PCET) occurs to generate key intermediates such as *COOH, *CO, and *OCHO. These intermediates then undergo subsequent coupling and desorption to form target products [8]. The final product distribution is determined by the transformation pathways of these intermediates, with the catalyst playing a critical role in regulating their adsorption strength. Strong adsorption may hinder product desorption and cause catalyst deactivation, while weak adsorption may fail to stabilize intermediates and maintain reaction efficiency [9,10,11]. Notably, the carbon–oxygen double bond in CO_2_ exhibits high stability, characterized by a bond dissociation energy of approximately 750 kJ·mol^−1^. This strong bond creates a significant kinetic barrier, resulting in a high overpotential requirement during the electrocatalytic CO_2_ reduction process. Additionally, the complexity of multi-electron transfer and proton-coupling steps severely limits target product selectivity [12,13,14]. Therefore, developing electrocatalysts with low overpotential and high Faradaic efficiency remains a major challenge in this field.

Transition metal-based catalysts exhibit differentiated performance in CO_2_RR. Au- and Ag-based catalysts are characterized by high selectivity for CO generation, which is achieved by promoting the stable formation of *COOH intermediates or enhancing the conversion of CO_2_ to CO on specific crystalline facets. Bi-based catalysts exhibit high affinity for CO2 chemisorption, which stabilizes the *OCHO intermediate and promotes efficient formate production. However, the products of these metal catalysts are mainly limited to the C_1_ species (e.g., CO and formic acid). After years of in-depth research, the potential for optimizing their catalytic mechanisms, structural regulation strategies, and performance has become relatively limited. With the growing demand for high-value utilization of carbon resources, research focus is shifting toward multi-carbon products, which have a higher energy density and greater industrial value. Against this background, Cu-based catalysts exhibit unique advantages in CO_2_RR and have become a central focus of current research. Notably, Cu is the only metal capable of efficiently converting CO_2_ into a range of hydrocarbons and alcohols with high selectivity [15,16]. A primary advantage of this process is its tendency to promote the formation of the critical intermediate *CO through the initial two-electron reduction step, rather than producing formate [17,18]. Additionally, its adsorption free energy for *CO (Δ*G*(*CO) ≈ 0.45 eV) falls within an ideal range, which not only stabilizes intermediates and promotes subsequent C−C coupling reactions but also avoids inactivation of active sites caused by excessive adsorption [19,20,21,22]. Hori et al. [23] studied the catalytic behavior of multiple metal electrodes in CO_2_RR and clarified the correlation between Cu electrode potential and product selectivity, thereby laying the foundation for research on Cu-based CO_2_RR catalysts.

Notably, Cu-based catalysts face significant challenges in the practical application of CO_2_RR, primarily due to slight variations in intermediate adsorption energies that complicate selective regulation and the significant competition from the hydrogen evolution reaction (HER) [24,25,26]. To address these challenges, catalyst structure, morphology, and surface electronic properties are being actively optimized by researchers. These efforts lay a foundation for modulating the adsorption behavior of reaction intermediates and improving catalytic selectivity. Recent investigations have revealed that adjusting the crystal facet exposure ratio of Cu significantly enhances C_2+_ product pathways [27], while engineering single-atom Cu sites with specific coordination environments markedly improves CO selectivity [28]. In addition, the alloying strategy has also been demonstrated to fine-tune intermediate adsorption energies by modulating surface electronic structures, suppressing HER, and enhancing the efficiency of specific C_2_ products [29]. Furthermore, surface modification techniques are employed to modulate local reaction microenvironments, increase the concentration of reactants, and inhibit side reactions [30].

**Figure 1:**
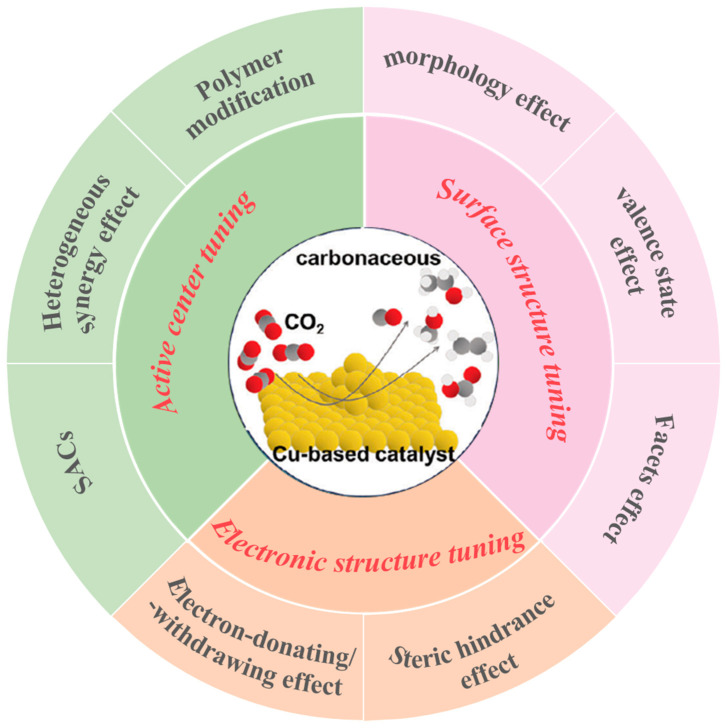
Scheme illustration of Cu-based catalysts design for CO_2_RR.

In summary, Cu-based catalysts are distinguished by their unique capability to produce multi-carbon products in CO_2_RR, attributed to their moderate adsorption energy for the key intermediate *CO. This characteristic establishes them as core materials for realizing high-value CO_2_ conversion [31]. However, challenges persist in their wider implementation, specifically the limited selective regulation of target products versus by-products, constrained by the moderate adsorption energy of key intermediates [32], and the broad product distribution arising from complex reaction pathways [33]. Fundamentally, these challenges stem from an insufficient understanding of dynamic changes in the surface electronic structure, active site density, and interfacial architecture of Cu-based catalysts during the reaction. Therefore, a deep understanding of the reaction mechanism and accurate modulation of Cu active site properties and their interactions with key intermediates are achieved through electronic effect tuning (e.g., electron-donating/-withdrawing effect [34], steric hindrance effect [35]), active center tuning (e.g., single-atom catalysts [36], heterogeneous synergetic effect [37], polymer modification [38]), and surface structure tuning (e.g., size/morphology effect [39], valence state effect [40], crystal facet effect [41]). This systematic approach constitutes the core pathway to enhance catalytic performance metrics, including activity, selectivity, and stability, as illustrated in Figure 1. This review aims to systematically synthesize the application advantages and catalytic mechanisms of Cu-based catalysts in CO_2_RR, with a focus on recent regulatory strategies derived from electronic effects and structural design, as well as interconnections. It seeks to offer a robust theoretical foundation and practical guidance for the rational structural and performance enhancement of Cu-based catalysts, thereby facilitating the practical implementation and advancement of high-efficiency CO_2_RR technology [42,43,44].

## 2. Electronic Structure Tuning

In electrocatalytic CO_2_ reduction, tuning the surface electronic configuration of catalysts is the core mechanism to overcome the bottleneck of product selectivity. Electron-donating/-withdrawing effects enable accurate reconstruction of electron cloud density and highest occupied molecular orbital (HOMO) symmetry at Cu active sites via ligand-induced conjugation synergy, thereby achieving directional modulation of the adsorption energy of *CO/*CHO intermediates [45,46,47]. Simultaneously, steric effects optimize the adsorption configuration entropy and reactant diffusion pathways through molecular topological constraints, cooperatively regulating the competitive pathways of C−C coupling and protonation. This dual mechanism ultimately dictates the activation energy barrier, reaction pathway, and product distribution of CO_2_ via atomic-scale electron–spatial coupling [48,49,50].

### 2.1. Electron-Donating/-Withdrawing Effect

**Figure 2:**
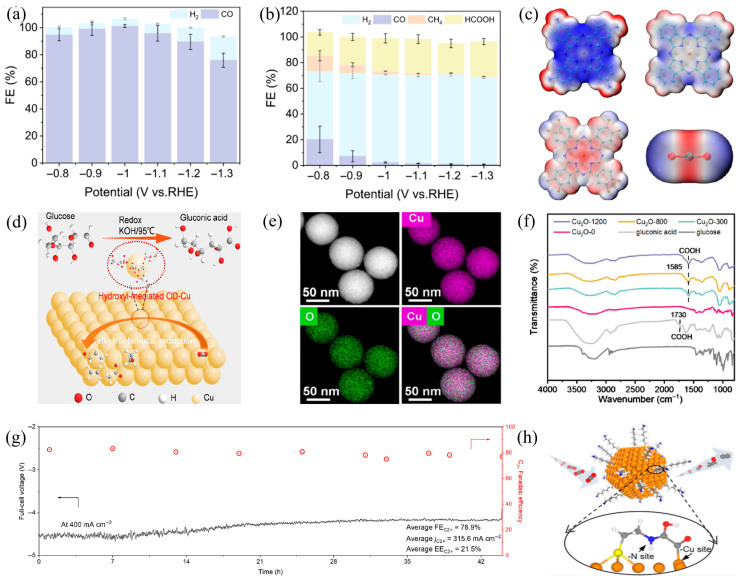
(**a**) The Faradaic efficiency of CuPc−NH_2_/CNTs, (**b**) The Faradaic efficiency of CuPc−F_8_/CNTs, (**c**) Electrostatic potential mapped van der Waals surface of CuPc−NH_2_, CuPc, CuPc−F_8_, and CO_2_ at the isosurface value of 0.2 a.u, (**d**) Schematic diagram of the action mechanism of surface hydroxylated Cu_2_O catalyst, (**e**) EDX elemental mapping of hydroxyl−functionalized Cu_2_O catalysts, (**f**) ATR−FTIRS spectra of as-prepared Cu_2_O−0, hydroxyl−functionalized Cu_2_O catalysts, glucose, and GA, (**g**) Full−cell voltage and C_2+_ product FE of Cu PTFE/C−F at 400 mA cm^−2^. The selectivity of C_2+_ products corresponds to the right vertical axis. (**h**) Schematic diagram of the aminosulfate ligand−Cu interface through organic (nitrogen (N)) and inorganic (Cu) interfacial active sites.

Electron-donating/-withdrawing effects enable fine regulation of electron cloud density at Cu active centers through synergistic inductive-conjugative coupling. This mechanism modifies the adsorption configuration, activation energy barrier, and subsequent reaction pathways of CO_2_ on the catalytic surface, enabling precise regulation of product selectivity in the electrocatalytic CO_2_ reduction process [51]. For instance, Fang et al. [52] synthesized two Cu phthalocyanine derivatives with opposing electronic properties: electron-donating group-modified CuPc−NH_2_ and electron-withdrawing group-modified CuPc−F_8_. Compared to unmodified CuPc, CuPc−NH_2_ significantly enhances CO_2_RR activity, achieving 100% Faradaic efficiency for CO in Figure 2a. Conversely, CuPc−F_8_ promotes the hydrogen evolution reaction, inhibiting CO_2_ reduction activity and yielding formic acid as the primary product in Figure 2b. Investigations reveal that ligand electron-inductive effects modify the surface electrostatic distribution of active central Cu atoms and regulate both the symmetry and spatial orientation of the HOMO through controlled Cu-ligand coordination in Figure 2c.

Li et al. [53] proposed a general strategy based on electron-donating effects, utilizing amino acid modifiers on Cu electrode surfaces to achieve selective electroreduction of CO_2_ into hydrocarbon products in Figure 2d. Studies show that, regardless of Cu electrode morphology, modified electrodes significantly enhance hydrocarbon generation efficiency via the electronic effect of amino acids in Figure 2e. Specifically, the amino group (−NH_2_) in amino acid molecules acts as an electron modulator, donating electron density to Cu active sites via an inductive effect, thereby adjusting the electron cloud distribution of the Cu centers. Density Functional Theory (DFT) calculations confirm that the key *CHO intermediate can be stabilized via electronic interaction with the positively charged −NH_3_^+^ group in adsorbed zwitterionic glycine. This electronic coupling effect, derived from the electron-donating nature of amino groups, effectively strengthens intermediate adsorption in Figure 2f.

Additionally, to further expand the role of electron-donating groups in optimizing Cu-based catalysts in CO_2_RR, Yu et al. [54] developed a copper porphyrin catalyst (CuTAPP) with a donor–acceptor structure. In this catalyst, amino groups (−NH_2_) act as electron donors to enrich the electron density at the central CuN4 active sites. This structural modification narrows the HOMO−LUMO gap to accelerate electron transfer, reduces the free energy barrier of the *CO−to−*CHO rate-determining step, and ultimately achieves a high Faradaic efficiency of 54.8% and a partial current density of 290.5 mA cm^−2^ for CH_4_ at −1.63 V (vs RHE). Complementing this molecular catalyst design, Wang et al. [55] investigated the modulation of the Cu (100) surface electronic structure by functionalized ionic liquids using Density Functional Theory. Their results showed that polar electron-donating groups (e.g., −SH, −COOH) in ILs transfer electrons to the Cu surface, upshift the Cu d-band center to strengthen *CO adsorption, lower the activation energy of *CO−*CO coupling, and suppress the HER by increasing the energy barrier of the HER rate-determining step. In contrast, non-polar −CH_3_ groups exerted negligible positive effects on CO_2_RR performance.

Furthermore, electron-withdrawing groups optimize electrocatalytic CO_2_ reduction performance by regulating electronic interactions between catalyst supports and active centers. For example, Wang et al. [56] reported a F-doped carbon-supported Cu catalyst (F−Cu/C). The electron-withdrawing effect of F induces electron cloud shift from the carbon support to F, creating an electron-deficient support. This electron-deficient state restructures the electronic configuration of the Cu active sites and reduces the activation energy required for C−C coupling, resulting in 82.5% Faradaic efficiency for C_2+_ products at 400 mA·cm^−2^ and 44 h of stability in Figure 2g. During electrocatalytic CO_2_ reduction, the electron-deficient support environment enhances *CO intermediate adsorption on Cu active sites. Simultaneously, the electron-withdrawing effect of F rearranges electron clouds between adjacent *CO intermediates, reducing resistance to C−C bond formation and promoting C_2+_ product formation. This demonstrates an effective strategy for enhancing CO_2_ electroreduction performance via support electron effect regulation.

Enhanced CO_2_ reduction performance of Cu-based catalysts is achieved by modulating electron transfer efficiency between donors and acceptors. For instance, Wan et al. [57] developed an aminothiol ligand-modified Cu catalyst, systematically investigating how ligand structure impacts CO_2_ reduction performance through the construction of stable organic–inorganic interfaces. The amino group in aminothiol acts as an electron donor, forming electronic coupling with copper active centers as electron acceptors through strong N−Cu bonds. The donor transfers electrons to the copper surface, which significantly enhances its electron density and provides sufficient electrons for the initial activation of CO_2_. Simultaneously, the cooperative interaction between N atoms in the ligand and Cu surface sites modulates the electron-rich environment of the active centers and lowers the activation energy required for the C−C coupling reaction in Figure 2h.

Similarly, extending the application of electron-withdrawing effects, from heteroatoms on catalyst supports or ligands to metal–chalcogen bonds in catalyst active sites. Wei et al. [58] reported that Sb incorporation induces the electron-withdrawing effect of Sb−S covalent bonds. This effect tunes the Cu−S bond length, thereby modulating the electron density of Cu active sites in CuS. The optimized electron density strengthens the binding of the *HCOO intermediate, enabling a 72% Faradaic efficiency for formic acid. Complementing this work, Wang et al. [59] further revealed that SO_4_^2−^ in the Cu_2_OSO_4_@CuO catalyst acts as a sacrificial electron-withdrawing agent. During the CO_2_RR, SO_4_^2−^ is reduced to S^2−^; the residual S^2−^ downshifts the d-band center of Cu, balancing the adsorption strength of *CO and *COH intermediates and lowering the energy barrier for C−C coupling. This regulation achieves a Faradaic efficiency of 88% for C_2+_ products.

### 2.2. Steric Hindrance Effect

**Figure 3:**
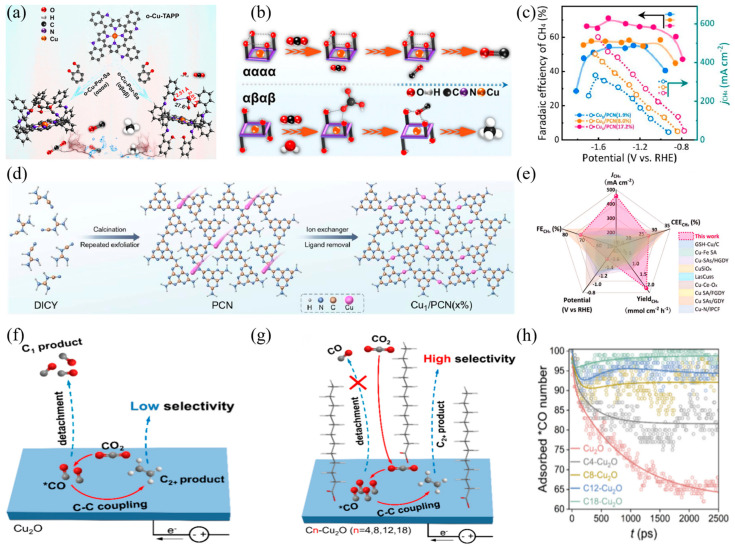
(**a**) CO_2_RR diagram of o−Cu−Por−Sa (αααα and αβαβ), (**b**) Schematic diagram of CO_2_RR catalytic mechanism for atropisomer o−Cu−Por−Sa (αβαβ, αααα), (**c**) Gibbs free energy diagrams of CO_2_RR on o−Cu−Por−Sa (αβαβ) and o−Cu−Por−Sa (αααα), (**d**) Schematic illustration of the fabrication of Cu_1_/PCN(x%) based on the periodic crystal structure of the PCN host, (**e**) Comparison of the performance metrics with the literature benchmarks for the CO_2_RR−to−CH_4_ conversion, (**f**) Illustrations show fast detachment of *CO intermediates on the pristine Cu_2_O surface, (**g**) Impeded *CO diffusion within the confined space constructed by long alkyl chains on modified C*_n_*−Cu_2_O surfaces, resulting in enhanced C_2+_ selectivity for CO_2_RR, (**h**) Simulated time profiles of adsorbed *CO numbers on Cu_2_O and different C*_n_*−Cu_2_O (*n* = 4, 8, 12, 18) surfaces.

In the electrocatalytic CO_2_ reduction system with Cu-based catalysts, steric effects are one of the key factors regulating product selectivity. Wang et al. [60] synthesized a sterically hindered porphyrin salicylimine Cu complex by adding hydroxyl groups to amino porphyrin ligands in Figure 3a. This complex catalyst uses hydrogen bonds from hydroxyl groups and the synergistic action of steric effects to precisely control the selectivity of CO_2_ reduction products in Figure 3b. Additionally, the symmetric distribution of hydroxyl groups around Cu active centers builds a specific spatial microenvironment. This steric hindrance not only enhances CO_2_ adsorption capacity but also drives the reaction pathway toward protonation by limiting intermediate diffusion. The Faradaic efficiency of CH_4_ reaches 84% in Figure 3c.

Similarly, Feng et al. [61] developed a periodic Cu single-atom catalyst using a sequential ion exchange strategy. By accurately anchoring Cu atoms to the periodic nitrogen sites of the polymeric carbon nitride (PCN) matrix, this strategy built low-valence Cu^δ+^ active centers with a diagonal coordination N−Cu−N configuration. With the synergistic effect of periodic N anchor sites in the PCN matrix and the delocalized π electron conjugation system, the catalyst can still achieve uniform dispersion of Cu atoms under high metal loading in Figure 3d. This unique steric arrangement limits the proximity of adjacent active sites, thereby inhibiting C−C coupling and blocking the reaction pathway for multi-carbon product formation. The optimized Cu_1_/PCN catalyst exhibited high selectivity toward methane in CO_2_RR. At −1.50 V (vs. RHE), a Faradaic efficiency of 71.1% for CH_4_ was achieved with a partial current density of 426.6 mA cm^−2^ in Figure 3e.

Different from the above strategy that uses steric hindrance to inhibit C−C coupling and enhance methane selectivity, the surface confinement strategy proposed by Li et al. [62] significantly enhances the selectivity of multi-carbon products in CO_2_RR by constructing a spatially confined microenvironment. This strategy achieves spatial confinement by grafting long-chain alkyl carboxylic acids (C_4_−C_18_) onto the surface of Cu_2_O nanoparticles in Figure 3f. The steric hindrance of long-chain alkyl groups effectively restricts the diffusion of *CO intermediates, prolongs their surface residence time, and raises the local CO concentration, thereby promoting the formation of multi-carbon products in Figure 3g. Experimental results confirm a Faradaic efficiency for C_2_H_4_ exceeding 63.0% on the C_12_/C_18_−Cu_2_O catalyst in Figure 3h. Furthermore, Han et al. [63] developed homogeneous Cu nanoclusters (Cu_13_ and Cu_14_) to precisely manipulate the electronic state distribution of Cu sites. This design lowers the adsorption energy of the *COOH on Cu_14_ surfaces, facilitating preferential CO_2_RR progression and achieving deep reduction to hydrocarbon products at −1.2 V (vs. RHE).

Unlike the aforementioned strategies that utilize steric hindrance to inhibit C−C coupling and enhance CH_4_ selectivity, recent studies on Cu-based catalysts have further expanded the regulation of steric effects to achieve multi-carbon product selectivity, with a variety of targeted approaches reported. Li et al. [64] grafted octadecyl chains with distinct terminal groups onto Cu nanowires; the steric hindrance of the long alkyl chains modulates the adsorption behavior of *CO intermediates and enhances the catalyst’s hydrophobicity to suppress the HER, ultimately achieving a Faradaic efficiency of 53% for C_2_H_4_.

Complementing this organic chain modification strategy, Huang et al. [65] synthesized Cu−Pd alloys, where Pd atoms induce a steric blocking effect to increase the spacing between adjacent *CO and *CHO intermediates, thus inhibiting C−C coupling, while adsorbing *H species to promote *CO hydrogenation, realizing a maximum Faradaic efficiency of 43.23% for CH_4_.

Esfandiari et al. [66] adopted a molecular immobilization approach, immobilizing Cu porphyrin on multi-walled carbon nanotubes; the steric confinement of the ultra-thin porphyrin layer restricts *CO diffusion, stabilizes the coexistence of Cu^2+^ and Cu^0^, and enables a Faradaic efficiency of 37.3% for CH_4_ with effective HER suppression.

At the cluster scale, Mohanta et al. [67] doped 3d/4d transition metal atoms into Cu_13_ clusters to form XCu_12_ bimetallic clusters; endohedral dopants induce the steric rearrangement of surface Cu atoms, which reduces the CO_2_ activation energy and lowers the overpotential for the CO_2_RR by approximately 20% compared to pure Cu_13_ clusters, laying a foundation for optimizing reaction kinetics via steric engineering.

Wang et al. [68] further manipulated steric effects at the nanosheet level by constructing Se vacancies in Cu_2−x_Se nanosheets; lattice stress-driven steric effects shorten the Cu−Cu spacing to 2.51 Å, thereby reducing the C−C coupling energy barrier and achieving a maximum Faradaic efficiency of 68.1% for ethanol.

## 3. Active Center Tuning

To address the limited selectivity toward multi-carbon products and industrial stability of Cu-based catalysts in CO_2_RR, catalyst material design has advanced toward multi-scale precise regulation. Single-atom catalysts can promote C−C coupling by utilizing high-density site configurations and dynamic structural reconstruction, thereby reducing the reaction energy barrier from a thermodynamic perspective. Bimetallic systems break the linear constraint of adsorption energy through interfacial functional design; they increase *CO coverage via d-band center displacement, promote C−C coupling, and improve multi-carbon product selectivity. Polymer catalysts are molecularly programmed to balance electron transfer and spatial confinement, achieving high total C_2+_ Faradaic efficiency at an industrial current density of 500 mA cm^−2^. These three material platforms, respectively, construct unique active centers with atomic dispersity, interfacial coordination, and hybrid dimensions, providing research directions for efficient C_2+_ synthesis.

### 3.1. Single Atom Catalysts

Single-atom catalysts (SACs) exhibit superior performance and maximized atom utilization in the electrocatalytic CO_2_ reduction, attributed to their atomically dispersed active centers, tailored electronic configurations, and unsaturated coordination environments [69,70,71]. Their core advantage lies in achieving atomic dispersion by the stable anchoring of isolated metal atoms on support surfaces, thereby combining the characteristics of homogeneous and heterogeneous catalysts. Even with metal atom utilization approaching the theoretical limit, SACs maintain good stability and recoverability, effectively enhancing catalytic activity and selectivity [72]. Notably, the stable existence of single-atom structures depends on strong interactions between supports and metal atoms, which effectively inhibit metal atom aggregation and migration [73]. With these distinct structural and performance advantages, SACs have shown great potential for selectively regulating multi-carbon products (especially C_2_ products) in CO_2_RR [74,75], consistent with the critical role of Cu-based catalysts in C_2_ product formation.

**Figure 4:**
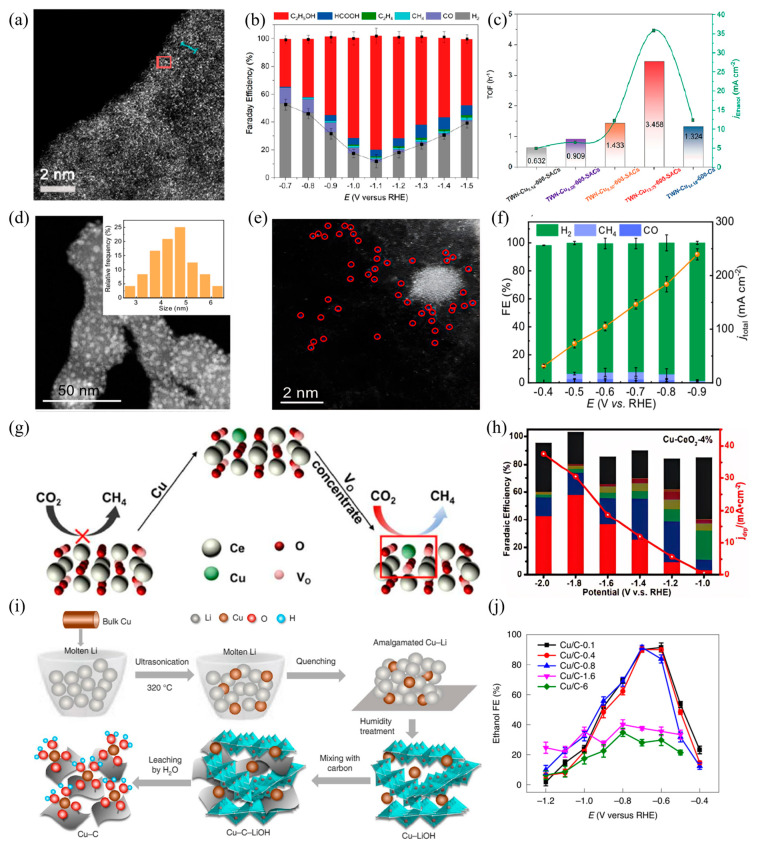
(**a**) HAADF-STEM images of TWN−Cu_13.35_−600−SACs, (**b**) The Faradaic efficiency and the product distribution at different potentials, (**c**) TOF, and partial current densities for ethanol conversion (blue line) tested at −1.1 V for TWN−Cu_x_−600−SACs and TWN−Cu_14.18_−600−Cs, (**d**) HAADF-TEM image (inset shows particle size distribution), (**e**) aberration-corrected HAADF-STEM image. The red circle marks the well-distributed Cu atoms in M-Cu_1_/Cu_NP_. (**f**) The Faradaic efficiency and total current density of Cu−N−C at different potentials, the data were averaged over three repeated measurements. (**g**) Schematic diagram of the reaction pathway, CO_2_ cannot be deeply reduced to CH_4_ on CeO_2_ catalysts. (**h**) The Faradaic efficiency (bars, left *y*-axis) and deep reduction products current density (j*_drp_*, red curves, right y-axis) of Cu−CeO_2_−4%, (**i**) Step-by-step preparation of the carbon-supported Cu SA catalyst using an amalgamated Cu−Li method, (**j**) The Faradaic efficiency of CO_2_−to−ethanol at different potentials over catalysts of different Cu loadings.

In the design of Cu-based SACs, active site density is a core parameter for regulating C_2_ product selectivity. High-density sites not only enhance catalytic activity per unit area but also promote C−C coupling via synergistic effects between adjacent sites. This mechanism is widely confirmed in C_2_ product formation over Cu-based catalysts [76,77]. Targeting this regulatory strategy, Xia et al. [78] prepared an ultrahigh-density Cu single-atom catalyst with a Cu component of 13.35 wt% on thin-walled N−doped carbon nanotubes in Figure 4a. Studies show that the high-density distribution of Cu−N_2_ sites is a key regulatory factor for ethanol selectivity. The short distance between adjacent Cu−N_3_ sites promotes C−C synergistic coupling, resulting in a Faradaic efficiency of 81.9% for CO_2_ conversion to ethanol and a partial current density of 35.6 mA cm^−2^ at −0.7 V (vs. RHE) in 0.1 M KHCO_3_ electrolyte saturated with CO_2_ in Figure 4b,c.

Density regulation of single active sites lays the foundation for C_2_ product formation, but the requirement for multi-step intermediate conversion in complex reaction pathways has driven the development of multi-active site collaborative systems. Dual active sites can simultaneously enhance intermediate generation and coupling steps through functional division. Thus, a dual active site coordination system has been developed. Feng et al. [79] synthesized a hybrid catalyst featuring synergistic active sites (M−Cu/CuNP) containing Cu nanoparticles and atomically dispersed Cu species anchored on an N-doped carbon matrix in Figure 4d,e. Experiments and theoretical calculations confirm functional division between the two sites: Cu nanoparticles promote *CHO dimerization, while atomic Cu sites facilitate hydrogen generation and transfer to accelerate *CO−to−*CHO conversion. Under their synergistic effect, the catalyst achieves a multi-carbon product Faradaic efficiency of 75.4% and a partial current density of 289.2 mA cm^−2^ in Figure 4f.

The synergistic effect of dual active sites depends on support regulation. Supports not only serve as anchoring matrices for active sites but also influence catalytic performance via their microstructure, which alters the spatial arrangement and electronic state of the sites. Yang et al. [80] systematically investigated the CO_2_RR performance of Cu single-atom catalysts (Cu−SAs) on carbon nanofibers (CNF) and tubular carbon nanofibers (TCNF). At −0.9 V (vs. RHE), the methanol Faradaic efficiencies of Cu−SAs/CNF and Cu−SAs/TCNF were 41% and 44%, respectively; this performance difference arises from distinct interactions between Cu single atoms and the two supports. Beyond macrostructural control of supports, the microenvironment surrounding active centers more directly affects CO_2_ adsorption and activation. Wang et al. [81] doped single-atom Cu onto CeO_2_ surfaces, such that each Cu site was accompanied by three oxygen vacancies, thereby constructing efficient CO adsorption and activation centers in Figure 4g. This structure endowed the catalyst with excellent methane selectivity, achieving a Faradaic efficiency of 58% in Figure 4h.

Notably, dynamic structural evolution (e.g., reversible aggregation or dispersion of sites) during electrocatalysis often goes unrecognized for its performance benefits, whereas dynamic reconstruction of Cu-based SACs under reaction conditions can optimize active site configurations in real time. Xu et al. [82] demonstrated performance gains from dynamic structures. The carbon-supported Cu−SAs prepared via a Cu−Li composite precursor route achieved a Faradaic efficiency of 91% for ethanol production at −0.7 V (vs. RHE) in Figure 4i. This performance enhancement arises from the synergistic interaction between metal clusters resulting from the dynamic aggregation of Cu atoms during the reaction and the remaining isolated Cu sites, which collectively facilitate C−C coupling by lowering the reaction energy barrier and optimizing the binding of key intermediates in Figure 4j.

### 3.2. Heterogeneous Synergy Effect

**Figure 5:**
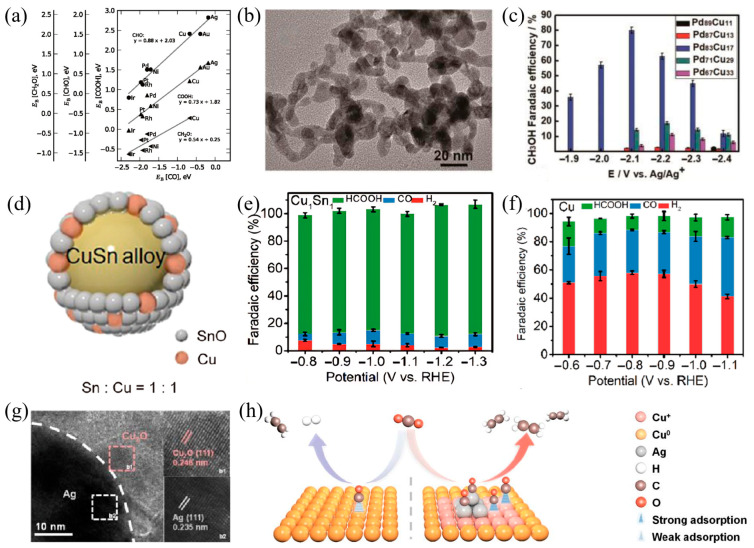
(**a**) Linear relationships between different reaction intermediates (scaling relationships), (**b**) TEM image of Pd_83_Cu_17_, (**c**) The Faradaic efficiency for CH_3_OH over Pd_x_Cu_y_ aerogels, (**d**) Schematic of Cu−Sn alloy, (**e**) The Faradaic efficiency of different products for Cu_1_Sn_1_ alloy, (**f**) The Faradaic efficiency of Cu, (**g**) HRTEM image of the Ag−Cu_2_O−0.10, (**h**) Schematic for enhanced C_2_H_4_ formation over Ag−Cu_2_O.

Cu is the sole metallic electrocatalyst capable of efficiently converting CO_2_ into multi-carbon compounds in the electrochemical reduction process, a capability primarily attributed to its distinct electronic configuration [83]. However, limited by the inherent linear relationship between intermediate adsorption energies, it is challenging to achieve synergistic optimization of activity and selectivity simultaneously. This leads to a broad product distribution and low efficiency of target products, severely restricting its industrial application [84] in Figure 5a. This linear correlation arises from the strong coupling between the adsorption energies of key intermediates. Such coupling implies that the adsorption strength of one key intermediate inevitably influences the binding behavior of correlated species, ultimately forming a thermodynamically driven bottleneck in product distribution. To address this constraint, Abild-Pedersen et al. [85] theoretically proposed an innovative approach: breaking the linear scaling relation by constructing Cu-based bimetallic catalysts through the introduction of a secondary metal, thereby leveraging multi-component synergy. Specifically, the electronic interaction can modify the adsorption strength of intermediates by tuning the d-band center energy level of Cu. For instance, the incorporation of Pd shifts the d-band center of Cu downward, which results in weakened *CO adsorption. The geometric effect can be achieved through regulating the coordination number and interatomic distances at active sites. Meanwhile, interfacial synergy establishes a distinct charge distribution across bimetallic boundaries, which promotes the localized enrichment of *CO intermediates and accelerates the kinetics of C−C coupling [86]. This theoretical framework provides a foundational guideline for the directional design of catalysts and drives the development of bimetallic systems for different target products. The accurate selection of the second metal and adjustment of the interface structure enable breakthroughs in CO_2_RR performance.

In the field of methanol-selective catalysis, studies by Lu et al. [87] demonstrate the effectiveness of bimetallic design in regulating oxygenated intermediate conversion. They prepared Pd−Cu bimetallic aerogels via template-free self-assembly, constructing a three-dimensional porous structure consisting of crystalline Pd nanochains and amorphous Cu in Figure 5b. This structure not only optimizes the adsorption energy of CH_3_O intermediates through the electronic effect of Pd but also enhances CO_2_ mass transfer efficiency via high specific surface area and hierarchical pore structures. At −0.8 V (vs. RHE) in 0.1 M KHCO_3_, the catalyst achieves a Faradaic efficiency of 80%, a partial current density of 31.8 mA cm^−2^, and stable operation during 100 h of continuous electrolysis in Figure 5c. This unalloyed structure avoids excessive CO binding caused by Pd−Cu alloying, confirming the superiority of coordinated regulation at bimetallic interfaces.

Formate is a key liquid product of CO_2_RR, and its high selectivity relies on stabilizing the *OCHO intermediate. The Cu−Sn bimetallic catalyst developed by Zhang et al. [88] achieves this by optimizing the Cu/Sn ratio. Introducing tin creates coordination active sites suitable for HCOO^−^ adsorption via geometric effects, while weakening strong adsorption of CO through electron transfer, thereby inhibiting competitive reactions. At −0.8 V (vs. RHE), the catalyst exhibits 92% selectivity for formate, with a partial current density of 28.5 mA cm^−2^ in Figure 5d,e. This reflects the significant impact of bimetallic ratio regulation on product selectivity in Figure 5f. Similarly, Li et al. [89] developed Cu−Bi bimetallic nanostructures, in which the electronegativity difference between Cu and Bi promotes electron transfer from Cu to Bi. This mechanism leads to the formation of bimetallic sites featuring electron-enriched surfaces. These surfaces stabilize *OCHO adsorbates and lower the energy barrier for their formation, thereby selectively promoting the electrocatalytic reduction of CO_2_ to formate.

For multi-carbon hydrocarbon products, the efficiency of C−C coupling is the core bottleneck limiting performance enhancement. The interface-optimized Ag−Cu oxide catalyst designed by Wei et al. [90] specifically addresses this issue. Through heterojunction engineering of Ag and Cu_2_O, partially reduced Cu^+^/Cu^0^ active sites are induced at the interface in Figure 5g. Electronic modulation induced by silver enhances the surface coverage of *CO intermediates, whereas synergistic effects at the bimetallic interface reduce the energy barrier for C−C coupling in Figure 5h. DFT calculations reveal that the interface configuration facilitates an ethylene selectivity of 66.0% and a partial current density of 429.1 mA cm^−2^, exceeding the performance of most documented Cu-based catalysts. This fully confirms the critical role of the interface effect in promoting the formation of multi-carbon products.

### 3.3. Polymer Modification

Polymer-modified Cu-based catalysts exhibit promising potential for improving C_2_ product selectivity during electrochemical CO_2_ reduction, due to their ability to precisely modulate the electronic properties and spatial arrangement of active sites [91]. The polymer modification strategy enables dual precise regulation via coordination between ligands and Cu centers. On one hand, heteroatoms in the polymer tune the d-band center of Cu through electron transfer, thereby optimizing the adsorption strength of critical intermediates including *CO and *CHO. On the other hand, the spatial confinement effect of the polymer skeleton enables precise control over the spacing between neighboring Cu active sites, providing a geometrically compatible environment for dual-intermediate coupling [92]. However, traditional polymer modifications often result in current densities below 100 mA cm^−2^ due to excessive wrapping of active sites or hindrance of CO_2_ mass transfer, failing to meet industrial demands for high current density [93]. These drawbacks have prompted researchers to develop Cu coordination polymers with stable structures, aiming to balance active site exposure and mass transfer efficiency through periodic skeleton design, ultimately enabling concurrent enhancement of both catalytic activity and selectivity.

**Figure 6:**
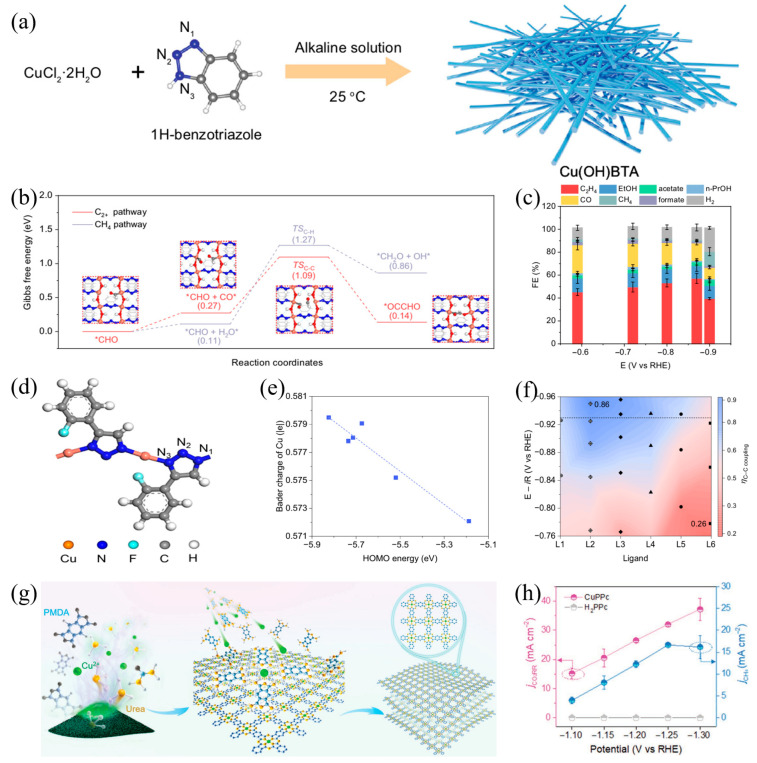
(**a**) Schematic illustration of the synthesis procedure, (**b**) Gibbs free energy diagram of CO_2_RR to C_2+_ and CH_4_ pathway on Cu(OH) TA slab. Inset figures: top-view geometries of intermediates (*CHO, *CHO+*CO, TSC-C, and *OCCHO) along CO2-to-C2+ pathway on Cu(OH) BTA slab. (**c**) The Faradaic efficiency of all products under different applied potentials over Cu(OH) TA in 1 M KOH electrolyte, (**d**) The Cu−N coordination models in the modeling, (**e**) The relationship between Bader charge of Cu in Ln−Cu and HOMO energy of the ligands. The lines is HOMO energy of ligands tunes Cu electronic states. (**f**) Colored contour map of η_C−C_ coupling. The coloured contour map showed the ηC–C coupling increasing from −0.76 to −0.96V in all Ln-Cu samples. (**g**) Schematic illustration of the synthesis and structure of CuPPc, (**h**) Total CO_2_RR current density and CH_4_ partial current density on CuPPc and H_2_PPc.

The mononuclear Cu coordination polymer Cu(OH)−BTA designed by Liang et al. [94] exemplifies this concept. Using 1,2,3−benzotriazole (BTA) as the ligand, the catalyst forms a periodic three-dimensional network via Cu−O and Cu−N coordination bonds in Figure 6a. Spatially adjacent Cu atoms in the polymer create geometrically compatible dual active sites, which can drive the formation of *OCCHO intermediates through a more energy-efficient pathway following the rate-determining step of CO hydrogenation in Figure 6b. In a flow electrolytic cell, the catalyst demonstrates remarkable C_2_ product selectivity at a current density of 500 mA cm^−2^, achieving a Faradaic efficiency of 73% for total C_2_ products and 57% specifically for ethylene in Figure 6c, resolving the mass transfer limitation of traditional polymer-modified catalysts (typically <100 mA cm^−2^) via balanced active site exposure and conductivity.

Yang et al.’s research [95] further reveals how ligand electronic structure regulates catalytic performance. They used six phenyl−1H−1,2,3−triazole derivatives to coordinate with Cu ions, constructing stable mononuclear Cu coordination polymers. Adjacent Cu atoms in the polymers provide an appropriate distance between dual Cu sites in Figure 6d, facilitating the formation of *OCCHO intermediates after the rate-determining step of CO hydrogenation. By adjusting the HOMO energy level of the ligands, the electron density of Cu active centers and their binding energy to *CO intermediates can be directionally regulated. This enables wide-range control of C−C coupling efficiency, with values ranging from 0.26 to 0.86 in Figure 6e,f.

Notably, the polymer modification strategy is not limited to C_2_ products; it is equally effective for selective regulation of single-carbon products. Zhang et al. [96] prepared a conjugated Cu phthalocyanine polymer (CuPPc) catalyst via solid-state synthesis, which exhibits excellent methane selectivity in CO_2_RR. Studies show that spatially isolated Cu−N_4_ active sites in CuPPc effectively inhibit C−C coupling, promote *CO intermediate protonation, and stabilize the vital *CHO intermediate, thereby improving the selectivity of the CH_4_ generation pathway in Figure 6g. At −1.25 V (vs. RHE), the Faradaic efficiency of CH_4_ reaches 55% with a partial current density of 18 mA cm^−2^ in Figure 6h. This result demonstrates that directional conversion from C_1_ to C_2_ products can be achieved by adjusting the porosity and active site spacing of the polymer skeleton, reflecting the universality of the polymer modification strategy.

## 4. Surface Structure Tuning

For industrial translation, surface structure tuning is a key engineering strategy for achieving high-efficiency CO_2_RR. The size and morphology effects optimize mass transfer pathways and active site exposure through the design of hierarchical nanostructures, thereby increasing the local concentration of *CO. The valence state effect, combined with static interface construction and dynamic in situ reconstruction strategies, modulates the density of d-band electronic states to reduce the C−C coupling barrier. The crystal facet effect regulates the C_1_/C_2_ reaction pathway by exposing specific crystal planes. Through cross-scale synergy, the three-dimensional control system establishes a regulatory strategy ranging from the optimization of nanoscale mass transfer microenvironments to the precise control of atomic-level reaction pathways.

### 4.1. Morphology Effect

The nanoscale morphology of Cu-based catalysts exerts a decisive influence on electronic structure and surface energy, thereby determining their catalytic activity and product distribution in CO_2_RR [97]. Given the crucial role of morphology in regulating catalytic performance, Cu-based catalysts have become a major research focus in the CO_2_RR field due to their diverse adjustable geometric configurations and excellent catalytic potential. A variety of nanostructured Cu-based catalysts, including dendritic forms, nanowires, nanorods, nanotubes, polyhedra, hollow porous spheres, and core–shell systems, have been successfully synthesized and meticulously characterized in recent studies [98].

**Figure 7:**
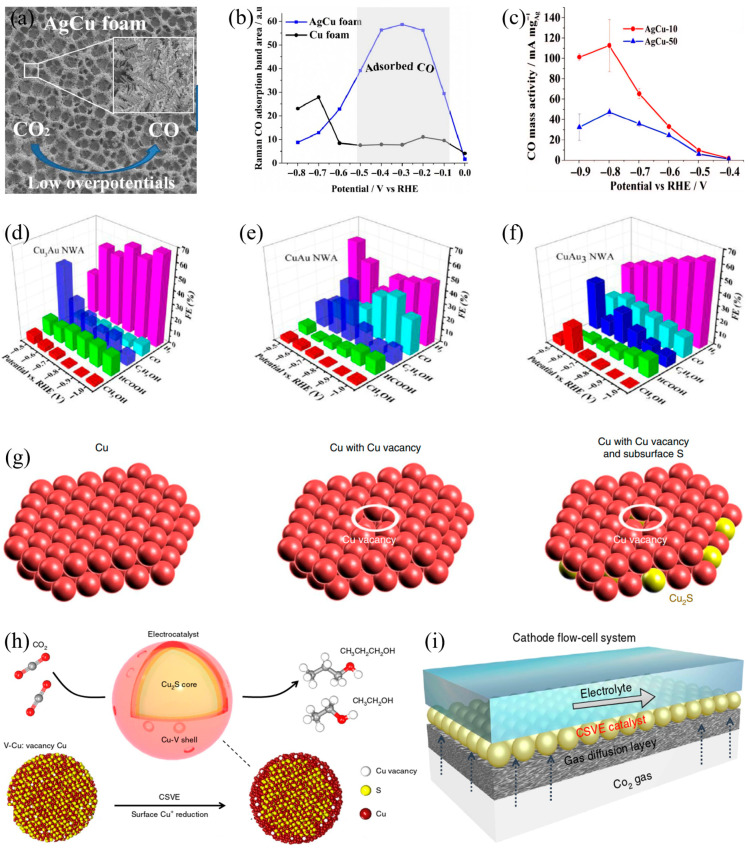
(**a**) This is a schematic illustration of AgCu foam used as a catalyst for CO_2_ electroreduction. The inset shows a magnified view. (**b**) A comparison chart of Raman spectra band area changes in CO adsorption on AgCu foam and Cu foam under different potentials (a.u is a basic unit). (**c**) CO mass activity with respect to Ag loading in AgCu−10 and AgCu−50, (**d**) The Faradaic efficiency at different potentials of Cu_3_Au NWA, (**e**) The Faradaic efficiency at different potentials of CuAu NWA, (**f**) The Faradaic efficiency at different potentials of CuAu_3_ NWA, (**g**) Atomic models of Cu_2_S−u−V, (**h**) Schematic illustration of Cu_2_S−u−V CSVE electrocatalyst design for production of multi-carbon alcohols from CO_2_ reduction (core–shell-vacancy engineering (CSVE)), (**i**) Schematic illustration of the cathode flow-cell system using a gas-diffusion electrode for CO_2_.

Among various nanomorphologies, nanodendrites feature a highly branched three-dimensional network structure and abundant edge-step sites, making them an ideal model for studying how morphology regulates CO_2_RR performance. This structure increases the density of active sites through a larger specific surface area, and at the same time, adjusts the energy barrier for intermediate adsorption through its complex surface topography, thereby optimizing the reaction pathway [99,100]. Kottakkat et al. [101] explored the effect of bimetallic Cu−Ag dendritic nanostructures on product distribution and selectivity. They found that introducing Ag during the electrodeposition process significantly changes the size and morphology of the dendrites formed in the pore walls of the Cu−Ag foam, thereby increasing the specific surface area and surface roughness of the catalyst in Figure 7a. These morphological modifications promote a greater availability of active sites, strengthen CO adsorption, and consequently elevate the current density of CO_2_ reduction, leading to improved overall catalytic efficiency in Figure 7b,c. Subsequent studies have shown that the needle-like structures at the edges of Cu−Ag dendrites increase the local pH at the electrocatalytic interface, which in turn promotes the selective production of C_2_H_4_ while inhibiting the formation of CH_4_, thereby optimizing the product distribution of CO_2_RR.

Beyond nanodendrites, one-dimensional (1D) nanostructures (e.g., nanowires) exhibit exceptional CO_2_RR performance, primarily attributed to their unique electron transport pathways and mass transfer efficiency. The high aspect ratio of 1D structures enables rapid charge transfer and adjusts ion diffusion in the electrolyte through the confinement effect, thereby optimizing the reaction microenvironment [102]. Cu_x_Au_y_ nanowire arrays (NWAs) were synthesized by He et al. [103] for CO_2_RR. Experimental results demonstrated that the NWA structure restricts OH^−^ and HCO_3_^−^ diffusion, leading to increased local pH and enhanced *CO intermediate coupling. Additionally, this architecture reduces CO desorption from the catalyst surface to the bulk solution, enriches the local CO concentration at the surface, and elevates the *CO/*H ratio, collectively enabling highly selective ethanol generation at lower potentials in Figure 7d–f.

Compared with simple one-dimensional morphologies, core–shell structures provide a new strategy for optimizing the performance of Cu-based catalysts by introducing synergistic effects at heterogeneous interfaces. Electron interactions between the core and shell modulate surface electron density, while precise tuning of shell thickness controls intermediate adsorption strength to achieve high product selectivity. Zhang et al. [104] fabricated Cu@Ag core–shell nanoparticles with precisely tunable Ag shell thickness. The Cu@Ag−2 catalyst, with an optimally designed Ag shell, exhibited the highest Faradaic efficiency of multi-carbon products. It was found that an appropriate Ag shell enriches *CO on the surface of the Cu core, while the core–shell structure provides a higher density of active sites and accelerates charge transfer dynamics, which together improve the conversion efficiency of CO_2_ to C_2_ products.

Building on these structural designs, defect engineering offers more precise control to achieve breakthroughs in the performance of Cu-based catalysts. By introducing vacancies or heteroatoms into the core–shell structure, the electronic properties and surface energy of the catalyst can be precisely adjusted, leading to customized reaction pathways and the inhibition of competing side reactions [105,106]. Zhuang et al. [107] proposed a new Cu-based catalyst with core–shell vacancy engineering. This catalyst features sulfur atoms anchored within the nanoparticle core along with Cu vacancies in the shell, enabling precise modulation of the local electronic structure of the catalyst in Figure 7g. In a flow electrolytic cell, the catalyst exhibited outstanding performance at 500 mA cm^−2^, achieving a total Faradaic efficiency of 32% for C_1_ alcohols and 57% for C_2_H_4_, representing 1.3-fold and 1.5-fold enhancements, respectively, over conventional Cu catalysts in Figure 7h. This performance enhancement arises from the sulfur-doped core modulating the electronic structure of the Cu shell, redirecting the protonation pathway of *CO intermediates from ethylene formation to alcohol production, thus explaining why vacancy engineering alters product pathways. Furthermore, in a membrane electrode assembly configuration, the catalyst demonstrated stable operation for over 67 h in Figure 7i.

### 4.2. Valence State Effect

**Figure 8:**
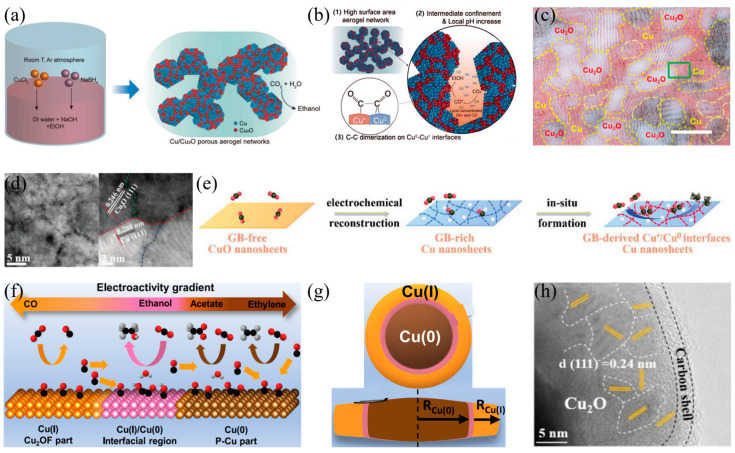
(**a**) Synthesis and structure of the Cu/Cu_2_O aerogel, (**b**) Rationale for the improved electrochemical ethanol synthesis performance on Cu/Cu_2_O aerogel electrodes, (**c**) HRTEM image with inverse-FFT mapping of b−Cu_2_O/Cu. The yellow highlight indicates the Cu_2_O nanodomain. (**d**) High-resolution HAADF−STEM image with magnified region of GBs and Cu^+^/Cu^0^ interfaces, (**e**) Schematic illustration of the reconstruction mechanism of CuO−160W under CO_2_RR condition, (**f**) Schematic illustrations of the reaction mechanisms on P-Cu_x_/Cu_2_OF, (**g**) The Cu(I)/Cu(0) core/shell nano disk for P−Cu_1.65_/Cu_2_OF, (**h**) The HRTEM images of CuO@C. The yellow highlight indicates the lattice spacing of Cu_2_O.

In CO_2_RR, tuning the valence states of Cu-based catalysts significantly affects product selectivity. Different Cu valence states (e.g., Cu (I), Cu (II), and Cu (0)) directly change the surface electronic structure of the catalyst, which in turn influences its interaction with reaction intermediates and ultimately determines the CO_2_ reduction pathway and product distribution [108]. This characteristic has made Cu-based catalysts a focus in research on converting CO_2_ into high-value-added chemicals. Researchers modulate the valence state distribution of copper through diverse strategies to enable efficient generation of target products. Kim et al. [109] reported a Cu/Cu_2_O aerogel characterized by high specific surface area and a porous network structure, fabricated through a redox reaction between metal precursors and a reducing agent in Figure 8a. When applied to CO_2_RR, this material exhibited a Faradaic efficiency of 41.2% for ethanol at a partial current density of 32.55 mA cm^−2^. Studies showed that the widely distributed Cu^0^−Cu^+^ interfaces in the Cu/Cu_2_O aerogel and the increase in local pH during the reaction are key factors promoting the conversion of CO to ethanol in Figure 8b. Building on studies of Cu/Cu_2_O composite structures, Zhang et al. [110] proposed a bicontinuous b−Cu_2_O/Cu electrocatalyst (b−Cu_2_O/Cu). It consists of ultra-small Cu_2_O nanodomains, Cu nanodomains, and abundant grain boundaries between them in Figure 8c. This structure facilitates the formation of *CO, *CO−CO coupling, and *CO−*OCCO coupling. Thus, the Cu_2_O/Cu catalyst exhibited a Faradaic efficiency of 12.1% for CO_2_ reduction to propanol at −1.1 V (vs. RHE), with a partial current density of 101.6 mA cm^−2^.

In addition to regulating valence states by constructing specific composite structures, in situ reconstruction of catalysts during reactions has also become an important approach to modulate valence state distribution. Zhang et al. [111] prepared CuO nanosheets through electrodeposition with ultrasonic irradiation. Under CO_2_RR conditions, these nanosheets undergo structural reorganization, producing a high grain boundary density. The coordination-unsaturated Cu sites at these boundaries form stable Cu^+^/Cu^0^ interfaces which act as highly efficient active centers for CO_2_ conversion in Figure 8d. These interfaces elevate the surface coverage of *CO, lower the energy barrier for C−C coupling, and promote selective formation of C_2_H_4_ even at low overpotentials in Figure 8e. To further stabilize the Cu(I)/Cu(0) interface, Cai et al. [112] employed a CuF(OH) precursor to fabricate a fluorine-stabilized Cu(I) disk-like interfacial architecture (denoted as P−Cux/Cu_2_OF). This architecture comprises a Cu(I) shell encapsulating a zero-valent Cu core in Figure 8f. Experimental results show that the Cu(I)/Cu(0) interface region significantly enhances ethanol production, achieving a high C_2_ Faradaic efficiency of 80.2% in Figure 8g. Zang et al. [113] prepared a carbon-coated CuO_x_ (CuO_x_@C) catalyst via one-pot pyrolysis of a Cu-based metal–organic framework (MOF). This method effectively avoids the deep electroreduction of the Cu species and preserves the Cu^δ+^−Cu^0^ interface, thereby significantly reducing the energy barrier for the C−C coupling step, enabling a high C_2_ Faradaic efficiency of 82% in Figure 8h.

### 4.3. Crystal Facet Effect

**Figure 9:**
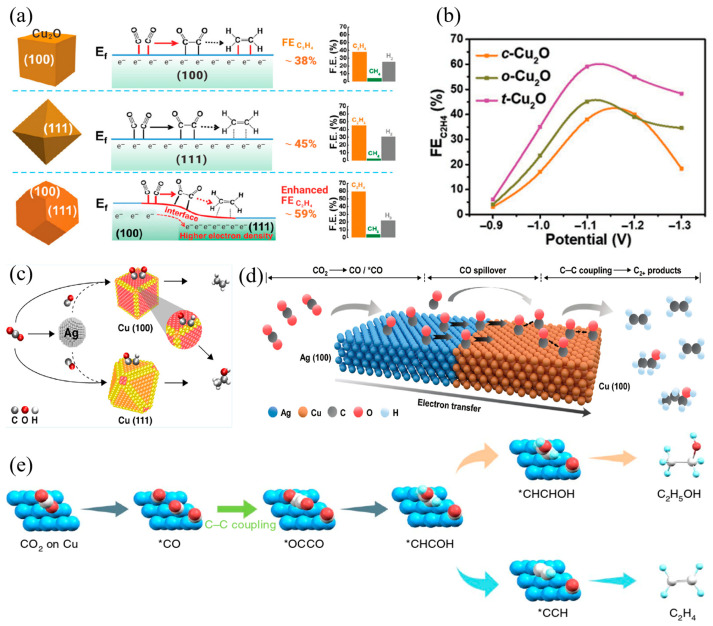
(**a**) Formation of C_2_H_4_ on Cu_2_O with (100), (111), and (100)/(111) mixed facets, (**b**) The Faradaic efficiency toward C_2_H_4_ for the c−Cu_2_O, o−Cu_2_O, and t−Cu_2_O NPs as a function of applied potential, (**c**) Schematic illustration of electrochemical CO_2_RR over Cu−Ag tandem catalysts, (**d**) Schematic illustration of a plausible CO_2_RR mechanism on Ag_65_−Cu_35_ JNS−100(6), (**e**) Schematic illustration of the mechanistic routes from CO_2_ to ethanol and ethylene through dimerization of two CO species. The * is the active site.

In the electrochemical CO_2_ reduction reaction, the crystal orientation of catalysts plays a decisive role in governing the adsorption energy and reaction pathways of key intermediates. This influence is exerted by changing the electronic properties and geometric configurations of active sites, which ultimately determines the distribution of reduction products. Hori et al. [114] first reported the structure–activity relationship between crystal planes and product selectivity in electrochemical CO_2_ reduction in 1995. Their results demonstrated that Cu-based nanocatalysts with different exposed crystal facets yield various carbon-containing products under identical conditions [115]. The close-packed Cu (100) crystal facets are more favorable for C_2_ product formation due to their low activation energy for C−C coupling: a conclusion further supported by Gao et al. [116] combined experimental and Density Functional Theory (DFT) study on Cu_2_O crystal facets. They found that, consistent with the behavior of metallic Cu (100), the Cu_2_O (100) crystal facet exhibits a strong affinity for CO adsorption, this adsorption preference, confirmed by DFT calculations, reduces the energy barrier for *CO dimerization by 0.3 eV compared to Cu_2_O (111) facets in Figure 9a. As a result, t−Cu_2_O achieves the highest C_2_H_4_ selectivity (up to 68%) among the three Cu_2_O phases, as shown in the selectivity comparison in Figure 9b. In contrast, Cu (111) crystal facets exhibit higher selectivity toward C_1_ products [117]. Thus, when designing high-performance Cu-based electrocatalysts, the crystal facet effect on catalytic performance must be comprehensively considered, whether for metallic Cu or Cu-based oxides like Cu_2_O, exposing (100) facets emerges as a viable strategy to enhance C_2_ product selectivity.

Building on the aforementioned basic research on the crystal facet effect, researchers have further explored strategies to enhance catalytic performance through crystal facet tuning and multi-component synergy. Iyengar et al. [118] investigated the impact of different crystal facets on CO_2_RR catalytic performance and proposed a series of catalytic strategies based on crystal facet regulation. In this study, Ag nanoparticles were loaded onto Cu catalysts with exposed specific crystal facets, constructing a series of bimetallic catalyst systems. For instance, nano-octahedrons with exposed Cu (111) crystal facets exhibit high selectivity for CH_4_ and are rich in CH_x_ intermediates. The Ag component provides a high concentration of CO species and promotes the formation of alcohol, which involves efficient coupling reactions between CH_x_ and CO in Figure 9c. On this basis, Ma et al. [119] prepared three different Ag−Cu heterostructure nanomaterials with dominant exposed Cu (100) facets. They achieved this by precisely adjusting the surface activity of the Cu precursor and the reduction kinetics, enabling site-specific epitaxial growth on the Ag substrate in Figure 9d. With the advancement of theoretical calculation techniques, researchers can analyze the intrinsic mechanisms of the crystal facet effect at the atomic scale. Li et al. [120] revealed the differential behaviors of Cu crystal facets under CO coverage through theoretical calculations. They found that, at high CO coverage, the key step following *CO dimerization is more likely to proceed via branching from the C_2_H_4_ pathway on Cu (111) crystal facets than on Cu (100) crystal facets in Figure 9e.

## 5. Conclusions

Cu-based catalysts exhibit significant potential in CO_2_RR, attributed to their unique electronic structure, excellent electrical conductivity, and moderate adsorption energy, which have made them a central research focus. This review summarizes the design strategies and mechanisms of Cu-based catalysts, emphasizing precise regulation of CO_2_RR product selectivity, activity, and stability through three dimensions: electronic structure tuning (electron-donating/-withdrawing effects, steric hindrance effects), active center tuning (single-atom catalysts, heterogeneous synergy effect, polymer modification), and surface structure tuning (morphology effect, valence state effect, crystal facet effect). These strategies enable effective regulation of electronic states at Cu active sites and adsorption behaviors of reaction intermediates, thereby enhancing the efficiency of CO_2_ conversion to high-value-added products and high selectivity for target products. However, challenges in practical applications remain, including insufficient structural stability, competition with the hydrogen evolution reaction, and an incomplete theoretical understanding for C_2+_ product formation pathways. Future research should prioritize addressing these challenges to optimize catalyst performance, while providing a theoretical foundation and practical reference for structural design and performance enhancement of Cu-based catalysts to advance the development of CO_2_RR catalysts.

Interface structure adjustment is pivotal for enhancing the catalytic performance of Cu-based electrocatalysts. Future research efforts should prioritize the comprehensive optimization of multi-scale interface structures, encompassing precise control over the size and morphology of nanoparticles, regulation of metal valence states, and engineering of exposed crystal facets. For instance, constructing interfaces between Cu^0^ and Cu^+^ species or designing nanostructures with specific facet orientations can markedly improve the selectivity toward multi-carbon products. Advanced in situ characterization techniques, including spectroscopy and microscopy are essential to gain deeper insights into the structure–performance relationships at these interfaces. Such fundamental understanding will provide critical guidance for the rational design of highly efficient catalysts. Furthermore, precise control of catalyst size and morphology enables the optimization of the specific surface area and the availability of active sites, leading to enhanced catalytic efficiency. This holistic optimization strategy not only boosts the intrinsic activity of the catalysts but also promotes their structural stability and operational durability under demanding reaction conditions.

Cu-based catalysts are prone to structural restructuring and valence state changes during reactions, leading to unstable performance. Future research should focus on addressing this issue by enhancing the corrosion resistance and anti-poisoning ability of catalysts through material design and surface engineering. For instance, developing catalysts with self-healing functions or designing highly stable porous structures can effectively improve the stability and service life of catalysts in practical applications. Additionally, introducing doping elements or constructing heterostructures can further optimize the electronic structure of catalysts and enhance their stability during long-term reactions. In-depth studies on the dynamic changes of catalysts during reactions will help reveal the mechanisms of performance degradation, providing theoretical support for designing more stable catalysts.

Theoretical calculations play a crucial role in catalyst design and reaction mechanism research. Future studies should further strengthen the integration of theoretical calculations and experimental research. Through computational methods such as density functional theory, the electronic structure of catalysts and the adsorption behavior of reaction intermediates can be predicted, providing theoretical guidance for experimental research. Meanwhile, advanced characterization techniques should be used to verify theoretical calculation results, enabling a deeper understanding of catalyst reaction mechanisms and precise regulation of catalyst performance. This combination of theory and experiment can not only accelerate the discovery of high-performance catalysts but also provides an essential theoretical basis for the optimal design of catalysts.

The translation of Cu-based catalysts from laboratory-scale research to industrial application requires greater attention to engineering applicability. Key challenges that must be addressed include enhancing catalyst durability at high current densities, scaling up synthesis methods, and ensuring compatibility with membrane electrode assemblies (MEAs) within flow electrolyzers. For example, developing gas diffusion electrodes (GDEs) incorporating stable Cu-based catalysts is essential to attain high current densities (>200 mA cm^−2^) and long-term stability (>1000 h)—both prerequisites for commercial viability. Furthermore, economic assessments and life cycle analyses should be incorporated early into the catalyst design process to evaluate both cost-effectiveness and environmental impact. Addressing these engineering-oriented issues will accelerate the translation of efficient CO_2_RR systems into practical deployments for sustainable carbon recycling.

## Data Availability

No new data were created or analyzed in this study. Data sharing is not applicable to this article.
